# A multicentre immersive virtual-world course for radiology residents: feasibility, educational outcomes, and participant perceptions

**DOI:** 10.1186/s13244-026-02455-6

**Published:** 2026-10-29

**Authors:** Alberto Pino-Postigo, Dolores Domínguez-Pinos, Eugenio L. Navarro-Sanchis, Miguel J. Ruiz Gómez, Francisco Sendra-Portero

**Affiliations:** 1 Department of Radiology and Physical Medicine, Facultad de Medicina, Universidad de Málaga, Málaga, Spain; 2 Department of Radiology, Hospital HLA El Ángel, Málaga, Spain; 3 Department of Radiology, Hospital Universitario Virgen de la Victoria Campus Universitario de Teatinos, Málaga, Spain; 4 IBIMA Plataforma BIONAND, Parque Tecnológico de Andalucía (PTA), Málaga, Spain; 5 Department of Radiology, Hospital Regional Universitario de Málaga, Málaga, Spain

**Keywords:** Radiography, Education (medical, graduate), Internship and residency, Virtual reality, Diagnostic imaging

## Abstract

**Objectives:**

To design and implement an immersive course on plain radiograph interpretation for radiology residents within a three-dimensional virtual world and to evaluate its feasibility, participants’ perceptions, and short-term changes in knowledge-test performance.

**Materials and methods:**

The course, delivered in a three-dimensional virtual world over 22 days, included seven 3-h synchronous sessions and asynchronous case-based activities. Educational outcomes were assessed using pre- and post-course multiple-choice tests. Participants’ experience was evaluated using 9-point Likert-scale items assessing cognitive load, perceived course level, and usefulness for clinical practice. After the course, participants completed an evaluation questionnaire including 5-point Likert-scale items, numerical ratings (0–10), and open-ended questions. Student’s *t*-test was used for continuous variables and the Mann–Whitney U test for ordinal data.

**Results:**

Sixty-seven residents from 27 hospitals met the certification criterion across two editions in 2021 and 2022. Forty-seven completed both the pre- and post-course tests, with mean scores increasing from 60.4 ± 6.5% to 70.2 ± 9.0% (*p* < 0.001). Participants reported positive perceptions, with high agreement for usefulness and adequacy (> 90%) and a mean usefulness score of 7.7 ± 1.2. Ratings were generally high (> 7/10), particularly for instructors, educational content, and course organisation, whereas interaction among participants received lower ratings.

**Conclusions:**

Immersive online teaching in a three-dimensional virtual world is a feasible approach for radiology resident education. Participation in the course was associated with improved short-term performance on a repeated knowledge test and positive participant perceptions. This format may complement traditional teaching methods in cohorts composed predominantly of early-year radiology residents.

**Key Points:**

***Question*** Flexible educational approaches are needed to reinforce plain radiograph interpretation training among radiology residents, but evidence supporting virtual-world training remains limited.

***Findings*** The virtual-world course was feasible and positively valued by participants, with participation associated with improved short-term performance on a repeated knowledge test.

***Critical relevance statement*** Immersive teaching in a three-dimensional virtual world is a feasible and well-accepted approach that may complement plain radiograph interpretation training in cohorts composed predominantly of first and second-year radiology residents.

**Graphical Abstract:**

**Figure:**
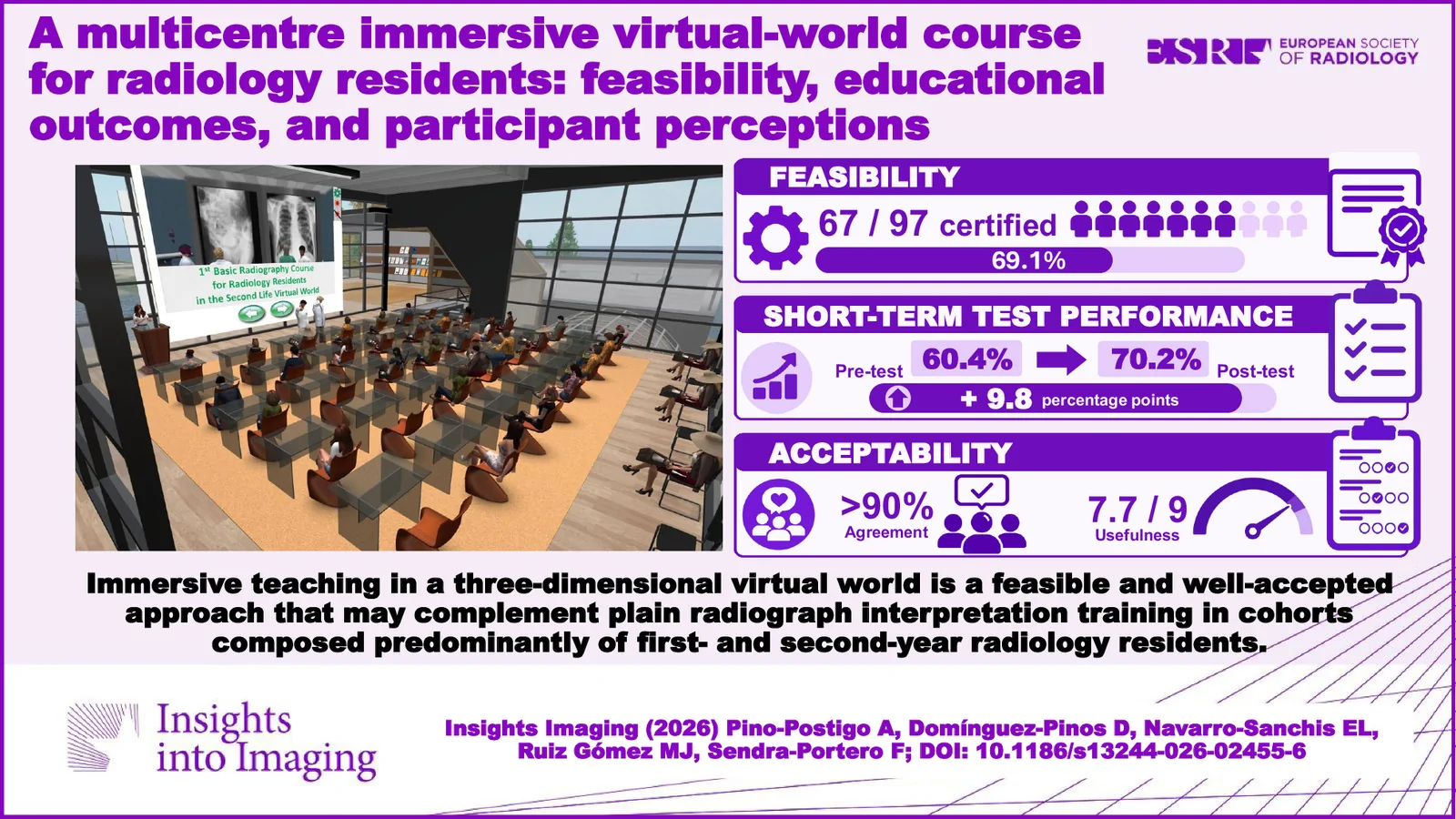

## Introduction

Despite the importance of diagnostic imaging in clinical practice, undergraduate teaching of plain radiology remains limited. Consequently, many first- and second-year radiology residents begin training with gaps in their knowledge, partly because of reduced opportunities for face-to-face hospital training during medical school. Increasing healthcare workload further limits teaching time in hospitals [1]. Although radiology residents are expected to interpret and report plain radiographs independently, only a minority have received adequate undergraduate training [2]. Moreover, few Spanish hospitals include dedicated rotations in plain radiography during radiology residency. Given the demand for plain radiograph interpretation in emergency departments and hospital wards, reinforcing these skills remains an important objective in the early years of radiology residency [3].

The expansion of online learning has increased interest in flexible digital teaching strategies for medical education [4, 5]. In this context, three-dimensional virtual worlds such as Second Life have been explored for synchronous and asynchronous educational activities. These platforms enable geographically distributed participants to interact through avatars in shared virtual spaces and have been widely used in healthcare education [6–9].

Virtual worlds are three-dimensional digital environments displayed on computer screens in which users interact through avatars, virtual representations of themselves [9]. Their educational use has increased in recent years [10], particularly in healthcare. Second Life, launched by Linden Research Inc. (San Francisco, CA, USA) in 2003, is one of the most widely used platforms in this field [11]. Previous studies have evaluated virtual worlds in several medical disciplines [12–16] and undergraduate radiology education [6–8, 17–19], demonstrating feasibility, positive learner perceptions, and educational outcomes comparable to conventional teaching. However, their application to postgraduate radiology training has not been reported, and their feasibility in this setting remains unknown.

Since 2011, Second Life has been used to deliver radiology educational activities [18]. Studies involving medical students have demonstrated the feasibility of synchronous and asynchronous teaching [7], positive learner perceptions [8], and educational outcomes comparable to traditional classroom teaching [6]. Building on these findings from undergraduate medical education, the present study extends this educational approach to postgraduate radiology training.

The primary aim of this study was to design, implement, and evaluate the feasibility of an immersive course on plain radiograph interpretation for first- and second-year radiology residents in a three-dimensional virtual world. Secondary aims were to assess participants’ perceptions and short-term changes in knowledge-test performance associated with course participation.

## Materials and methods

This prospective educational intervention included a pre–post assessment of knowledge outcomes and participants’ perceptions. Feasibility was evaluated through participant recruitment, attendance, certification, full course and assessment completion, technical usability, and participant acceptability.

### Study setting

**Fig. 1:**
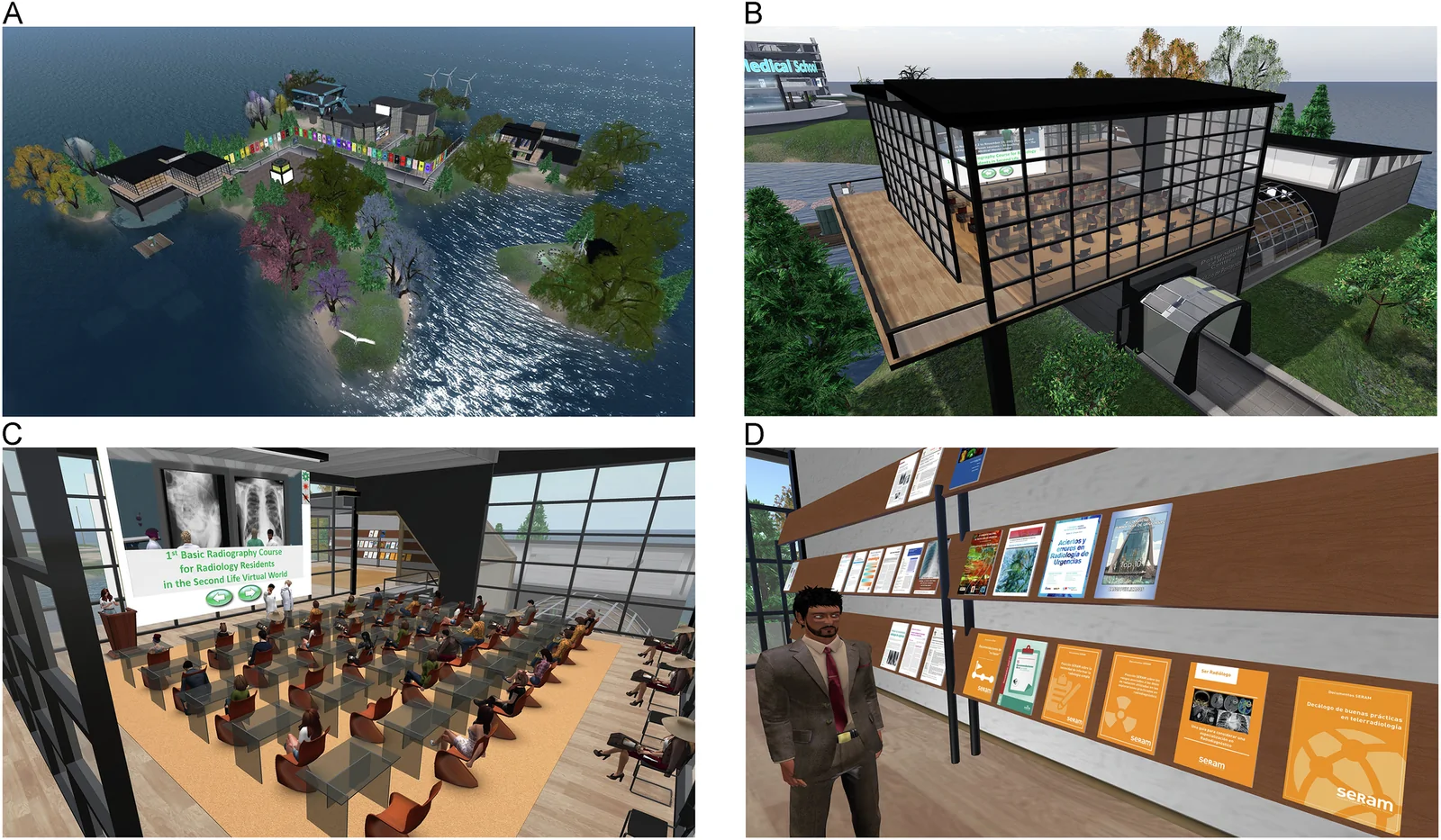
**A** Aerial view of the Medical Master Island virtual environment. **B** Postgraduate building where the course was conducted. **C** Scene during a synchronous session in the virtual classroom. **D** Virtual library; clicking on the displayed items allows downloading the content in PDF format

**Fig. 2:**
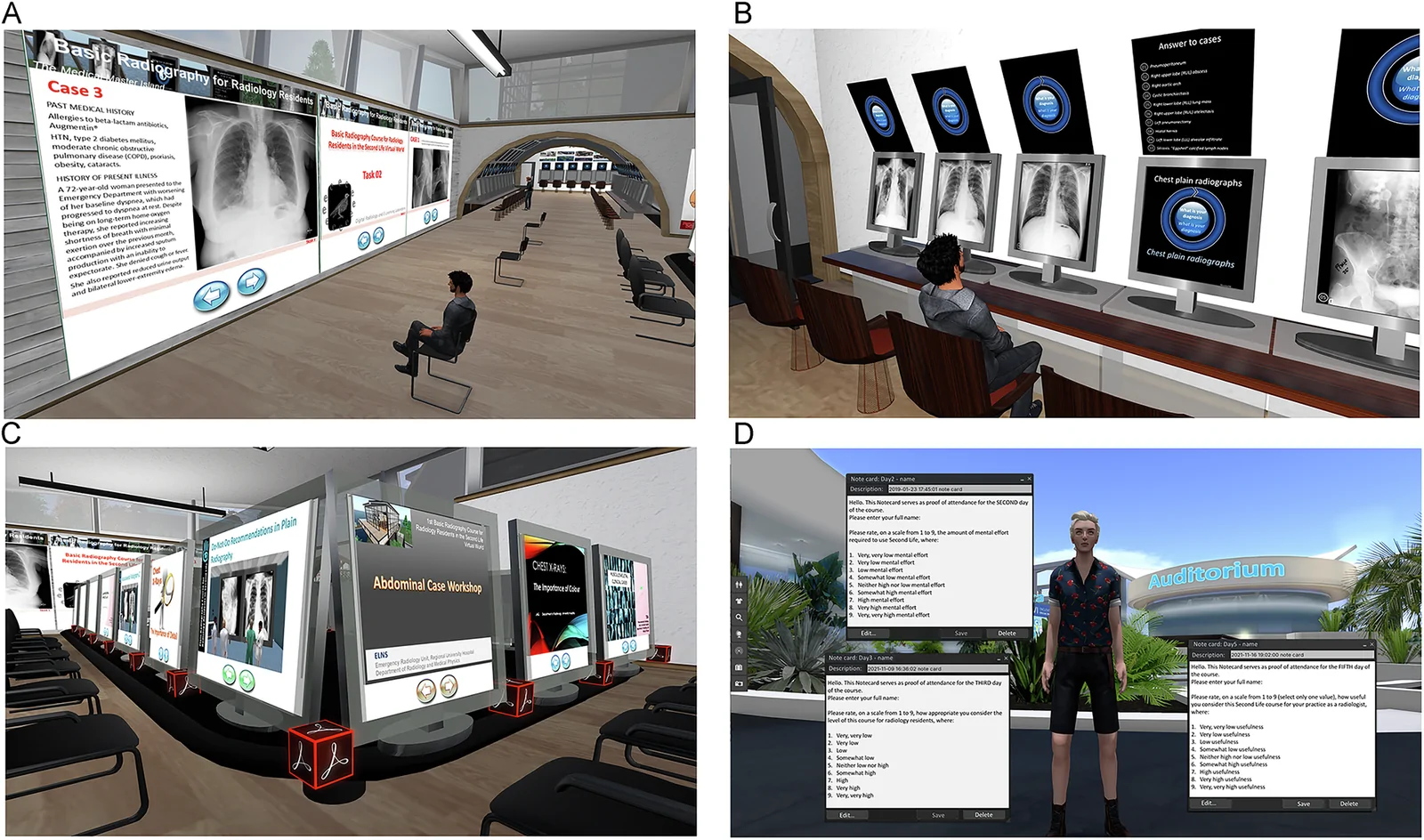
**A** Scene showing a resident completing asynchronous tasks displayed on wall posters, with three sets assigned per week. **B** Resident working on one of the virtual workstations, each containing 10 clinical cases. **C** Virtual workstations where recorded presentations from each instructor can be replayed, along with small cubes that allow downloading the presentations in PDF format. **D** Resident showing on screen the notecard system used to record attendance and to answer questions on a 9-point Likert scale

The Medical Master Island is a virtual environment resembling a university campus, with academic buildings connected by walkways and surrounded by vegetation (Fig. 1A). The postgraduate building used in this study (Fig. 1B) included a classroom for synchronous lectures and workshops (Fig. 1C), a virtual library (Fig. 1D), and an exhibition area with wall panels and virtual workstations for asynchronous activities (Fig. 2A, B) and providing access to course materials (Fig. 2C).

The course was delivered through the standard desktop version of Second Life, an avatar-mediated multi-user virtual world, without head-mounted displays or other virtual-reality hardware. Avatars can communicate through voice chat, written chat, and notecards. Voice chat enables real-time communication and a sense of presence, facilitating lectures, discussions, and debates. Written chat can be used for brief communications and participant questions. Notecards are in-world messages stored in users’ inventories for information exchange and assignment submission (Fig. 2D).

### Course design

We designed a 4-week course on plain radiograph interpretation in Second Life, delivered over 22 calendar days. Two editions were held from 9 to 30 November 2021 and from 1 to 22 February 2022. The course comprised seven 3-h synchronous sessions on Tuesdays and Thursdays, each consisting of three 1-h lectures, workshops, and case discussions (Table 1).

**Table 1:** Course programme

Day	Time	Content
Tuesday	17:00	Introduction to Second Life and basic platform training
	18:00	Importance of plain radiography in medical practice
	19:00	Initial evaluation
Thursday	17:00	Systematic interpretation of chest radiographs
	18:00	Systematic interpretation of musculoskeletal radiographs
	19:00	Systematic interpretation of abdominal radiographs
**Asynchronous tasks**		
Tuesday	17:00	Chest radiology I: Beyond the alveolar pattern
	18:00	Lower extremity radiography: common pitfalls
	19:00	Clinical cases in abdominal radiography
Thursday	17:00	Chest radiology II: Advanced chest radiograph interpretation
	18:00	Upper extremity radiography
	19:00	Evaluation of focal bone lesions on plain radiographs
**Asynchronous tasks**		
Tuesday	17:00	Chest radiology III: Chest radiography in emergency practice
	18:00	Spinal radiography
	19:00	Do-not-do recommendations in plain radiography
Thursday	17:00	Thoracic case workshop: attention to detail
	18:00	Musculoskeletal case workshop
	19:00	Abdominal case workshop
**Asynchronous tasks**		
Tuesday	17:00	Thoracic case workshop: the importance of grey levels
	18:00	Musculoskeletal case workshop
	19:00	Final evaluation

Ten senior radiologists participated as instructors. During the first session, participants visited the virtual island and classroom, received training in voice and text chat, notecards, and camera controls for image evaluation, attended an introductory lecture on plain radiography, and completed a pre-course test. Subsequent sessions focused on the systematic interpretation of chest, abdominal, and musculoskeletal radiographs through lectures, specific topics and clinical case discussions. Communication took place through voice and written chat, and participants submitted a notecard as proof of attendance (Fig. 2D). All synchronous sessions were recorded using OBS Studio (OBS Project, version 27.1.3) and remained available on demand for 1 month after course completion.

Participants completed two asynchronous tasks between sessions: three collections of clinical cases presented on wall posters (Fig. 2A), requiring a radiological report, and three sets of ten plain radiographs without clinical information displayed on virtual workstations (Fig. 2B), requiring diagnosis. Answers were submitted via notecards and discussed during the following session.

During the course, participants had access to presentations on virtual workstations and in PDF format (Fig. 2C), as well as bibliography and additional references in a virtual library (Fig. 1D). The course required approximately 30 h: 21 h of synchronous sessions and 9 h of asynchronous activities. A certificate of attendance was awarded to participants who completed more than 10 h.

### Participant enrolment

Participants were recruited through a PDF containing the course programme and participation instructions, disseminated via social media and the institutional channels of the Spanish Society of Medical Radiology (SERAM). A symbolic registration fee of €10 was charged, and enrolment was limited to 45 residents in the first edition and 50 in the second, on a first-come, first-served basis. Participation was therefore voluntary and may have favoured residents with greater motivation, availability, and access to adequate technology. After registration, participants received a welcome email and a PDF guide for using Second Life.

### Educational impact evaluation

During the first and final sessions of both editions, participants completed pre- and post-course tests consisting of 45 multiple-choice, single-best answer questions (15 per thematic block) selected by one author (FS-P) from the SERAM radiology question bank to match the course content. No formal psychometric evaluation of the selected questions was available. Questions were displayed sequentially on the virtual classroom screen, and participants had 30 s to answer each using Google Forms. Pre- and post-course responses were matched using participants’ email addresses, which were removed before analysis to ensure anonymity. Scores and correct answers were disclosed only after completion of the post-course test.

### Participants’ experience evaluation

During synchronous sessions, participants completed questionnaires using proof-of-attendance notecards (Fig. 2D). On the first and last days, they answered open-ended questions about their expectations and the course’s positive and negative aspects. On the remaining days, they completed 9-point Likert-scale items (1 = very, very low, 9 = very, very high; 5 = neutral) assessing mental effort to navigate Second Life (days 2, 4, and 6), perceived course level (day 3), and usefulness for clinical practice (day 5).

After the course, participants completed an evaluation questionnaire adapted from instruments used in previous studies [6–8, 17] to assess perceptions of Second Life and the course. It comprised: (1) a dichotomous question on prior familiarity with Second Life; (2) 5-point Likert-scale items assessing agreement with statements on Second Life (8 items), the course (11 items), and the chest, abdomen, and musculoskeletal presentations (12 items); (3) 0–10 ratings of eleven aspects of the project; and (4) open-ended questions on strengths, areas for improvement, and suggestions.

Participants were informed that the questionnaire data would be used exclusively to evaluate this educational experience. Data were recorded anonymously and stored in accordance with data protection legislation. Completion of the questionnaire implied informed consent. The educational activities described in this study were conducted in 2021 and 2022. Ethical approval for the project was granted by the local institutional ethics committee (approval No. 001-2023-H) on January 25, 2023, following review of the study protocol and the educational activities included in the project.

### Data analysis

Quantitative data were organised using Excel 365 (Microsoft, Redmond, WA, USA) and analysed using R (R Foundation for Statistical Computing, Vienna, Austria; version 4.4.2). Results were analysed separately by thematic block and course edition, excluding participants who completed only one test. Paired Student’s *t*-test was used to compare pre- and post-course test scores. Cohorts (2021 vs 2022) were compared using unpaired Student’s *t*-test for continuous variables and the Mann–Whitney *U* test for ordinal variables (Likert scales). A *p*-value < 0.05 was considered statistically significant.

Open-ended comments from the final evaluation questionnaire were analysed using an exploratory, inductive, descriptive approach. Responses were categorised as positive aspects, negative aspects, or suggestions for improvement, and all available comments were included.

## Results

### Participation

**Fig. 3:**
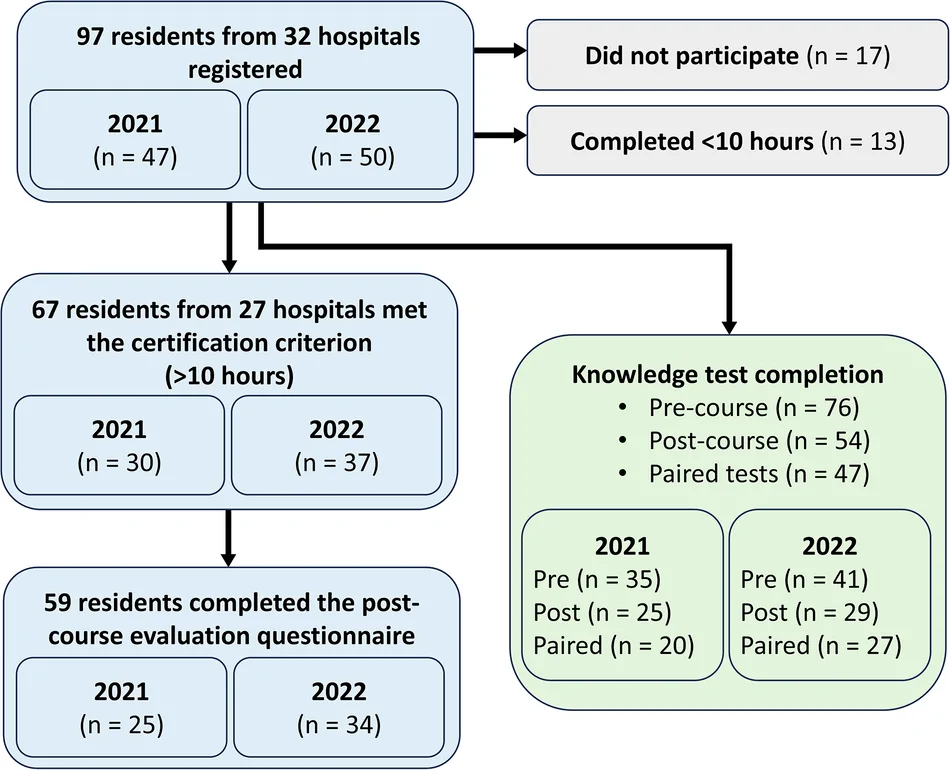
Participant flow diagram. Flow of participants through recruitment, course participation, certification, knowledge-test completion, and post-course evaluation questionnaire completion for the overall cohort and each course edition (2021 and 2022). Blue boxes indicate the course participation and evaluation pathway, green boxes indicate the knowledge-test pathway, and grey boxes indicate registered residents who either did not participate or completed fewer than 10 h

Ninety-seven radiology residents from 32 Spanish hospitals registered for the course (Fig. 3). Sixty-seven (69.1%) from 27 hospitals met the certification criterion (< 10 h of participation), including 28 men and 39 women. Among certified participants, 44 (65.7%) were first-year residents, 22 (32.8%) second-year residents, and one (1.5%) a third-year resident. Forty-seven completed 30 h, 12 completed 25 h, 7 completed 20 h, and 1 completed 15 h.

### Knowledge assessment outcomes

Pre- and post-course tests were completed by 47 residents (20 in the first edition and 27 in the second). Scores increased significantly in both editions and all thematic blocks, except for the thorax section in the second edition (*p* = 0.275). Mean total scores increased from 60.4 ± 6.5% in the pre-test to 70.2 ± 9.0%, corresponding to a mean paired difference of 9.8 percentage points (95% CI, 7.4–12.1; Cohen’s *d* = 1.22; *p* < 0.001), with significant improvements in all three thematic areas (Table 2).

**Table 2:** Results of the knowledge assessments expressed as percentages of correct answers

Edition	Contents	Pre-test	Post-test	*p*-value
First	Thorax	67.3 ± 14.5	79.7 ± 8.5	< 0.001
(*n* = 20)	Musculoskeletal	40.6 ± 8.2	51.5 ± 9.2	0.001
	Abdomen	65.0 ± 11.8	76.7 ± 7.0	< 0.001
	Total	57.3 ± 5.6	68.9 ± 6.2	< 0.001
Second	Thorax	71.9 ± 11.4	75.1 ± 13.6	0.275
(*n* = 27)	Musculoskeletal	45.4 ± 12.4	57.3 ± 16.9	0.001
	Abdomen	70.9 ± 8.7	81.2 ± 9.8	< 0.001
	Total	62.7 ± 6.3	71.2 ± 10.6	< 0.001
Both	Thorax	69.9 ± 12.9	77.0 ± 11.8	0.001
(*n* = 47)	Musculoskeletal	43.3 ± 10.9	54.7 ± 14.1	< 0.001
	Abdomen	68.4 ± 10.4	79.3 ± 8.9	< 0.001
	Total	60.4 ± 6.5	70.2 ± 9.0	< 0.001

Values are presented as mean ± SD. *p*-values were calculated using the paired Student’s *t*-test

### Evaluation of the experience

During-course 9-point Likert-scale questionnaires (5 = neutral) yielded the following results (Table 3). Mental effort required to operate in Second Life ranged from 4.7 ± 1.8 to 5.2 ± 1.7, indicating moderate cognitive load, and was slightly lower in the second edition. The perceived adequacy of the course level was 6.6 ± 1.1 (somewhat high to high), while perceived usefulness for clinical practice reached 7.7 ± 1.2 (high to very high). No significant differences were observed between cohorts, except for higher mental effort on day 4 in 2021 (5.7 vs 4.9, *p* = 0.042).

**Table 3:** Results of the evaluation notecards on cognitive load (mental effort), adequacy of the contents and usefulness of the course

Questions	1st edition	2nd edition	Both editions
How much mental effort does it take for you to get along in Second Life?			
Day 2	5.1 ± 1.7	4.7 ± 1.5	4.9 ± 1.6
Day 4	5.6 ± 1.5	4.9 ± 1.8	5.2 ± 1.7
Day 6	5.0 ± 1.0	4.4 ± 1.9	4.7 ± 1.8
How do you consider the level of this course for radiology residents?	6.8 ± 1.1	6.3 ± 0.9	6.6 ± 1.1
How useful is this course in Second Life for your practice as a radiologist?	7.7 ± 1.1	7.6 ± 1.3	7.7 ± 1.2

Answers were provided in a 9-point Likert scale: (1) Very, very low; (2) Very low; (3) Low; (4) Somewhat low; (5) Neither low nor high; (6) Somewhat high; (7) High; (8) Very high; (9) Very, very high

**Fig. 4:**
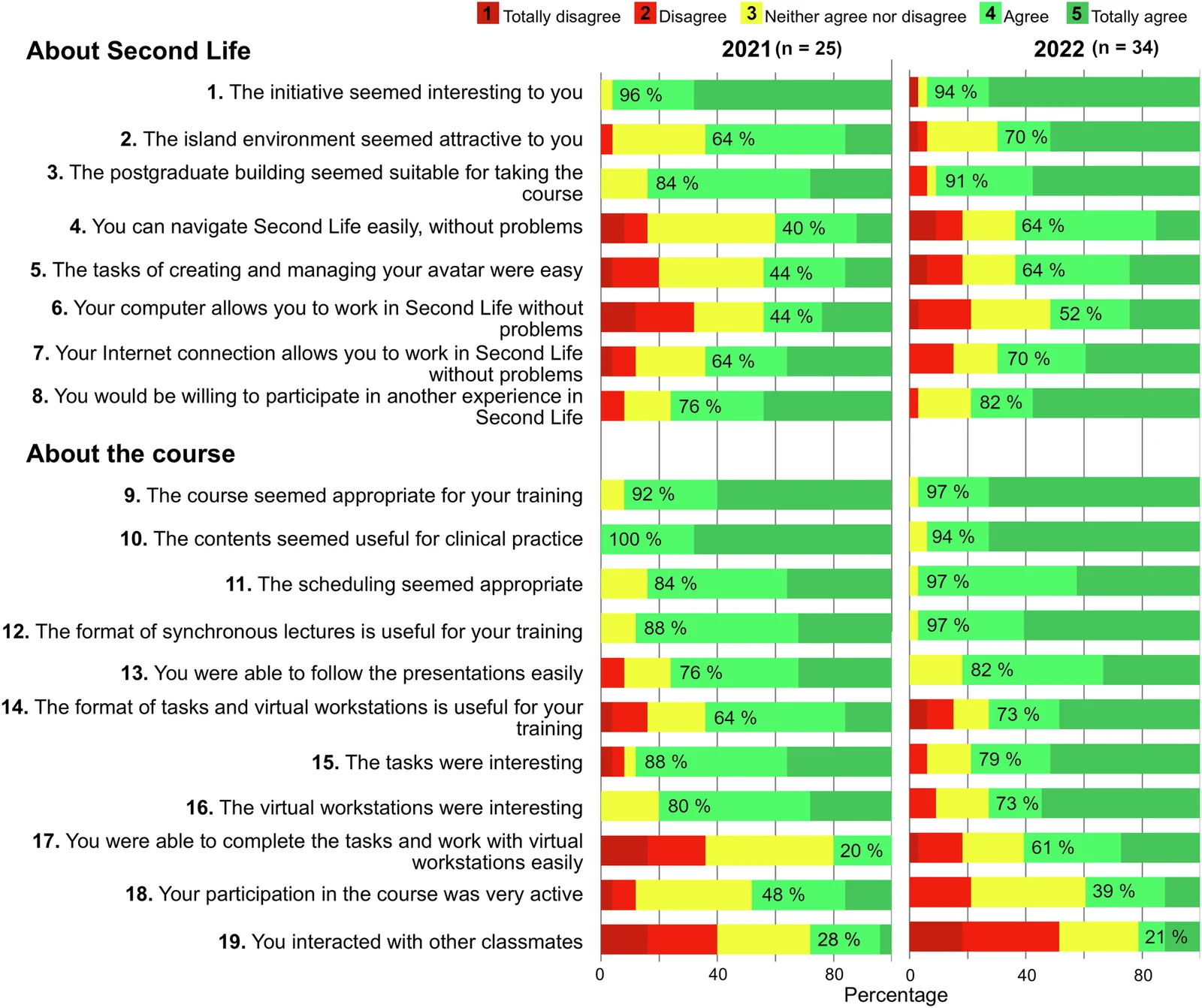
Bar chart showing the percentage distribution of responses to 19 statements about Second Life and the course on a 5-point Likert scale, comparing participants in 2021 (*n* = 25) and 2022 (*n* = 34). Red indicates disagreement, yellow neutrality, and green agreement. Values shown within the bars represent the percentage of participants reporting agreement (scores 4–5)

Fifty-nine residents completed the post-course evaluation questionnaire: 25 (83.3%) in the first edition and 34 (91.9%) in the second. Ten respondents (16.9%) were familiar with Second Life before the course. Likert-scale responses showed high agreement in both editions (Fig. 4 and Supplementary Table S1). For Second Life, agreement was highest for overall interest (94–96%) and willingness to participate again (76–79%). Ratings were also favourable for the virtual environment and infrastructure (64–91%), although lower agreement was observed for usability-related and technical aspects, including ease of use, avatar management, and hardware performance (40–64%). Agreement exceeded 90% for usefulness in clinical practice and adequacy for training and remained high for the synchronous format and course organisation (76–97%). Lower agreement was observed for task completion (20–61%), active participation (39–48%), and interaction among participants (21–28%). No between-cohort differences were observed, except for higher ratings in 2022 for synchronous lectures (*p* = 0.024) and task completion (*p* = 0.002).

Agreement with statements on the thoracic, abdominal, and musculoskeletal presentations was consistently very high. For usefulness, overall quality, and session clarity, agreement (scores 4–5) ranged from 88% to 98%. Agreement on the ability of the sessions to resolve doubts was slightly lower (75–82%) but remained high. The only significant between-cohort difference was higher agreement in 2022 for the clarity of musculoskeletal explanations (*p* = 0.024).

**Fig. 5:**
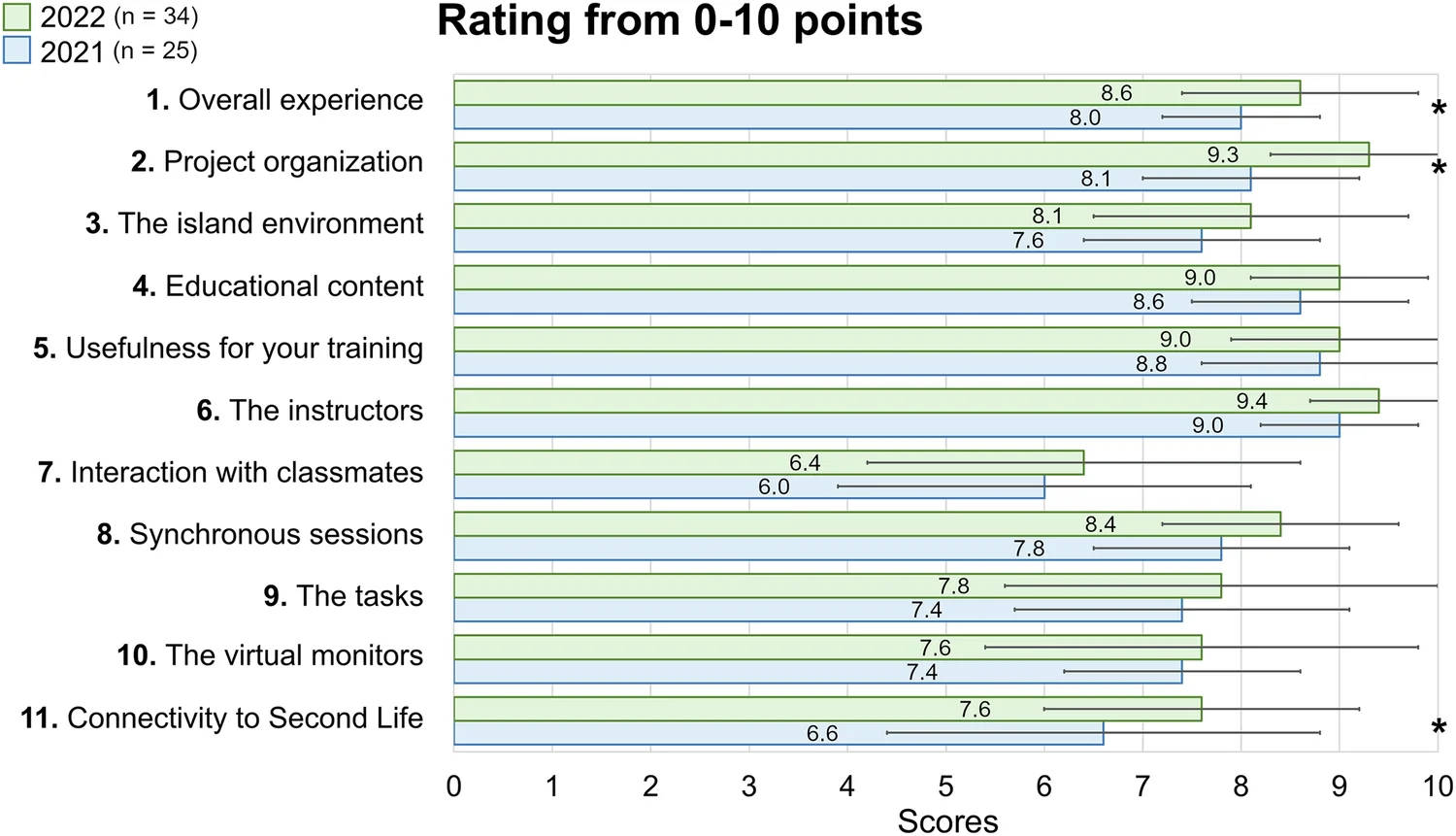
Bar chart showing the mean ratings (0–10 scale) of 11 aspects of the educational experience in 2021 (*n* = 25, blue bars) and 2022 (*n* = 34, green bars). Values shown within the bars represent the mean ratings, and error bars indicate standard deviation. Asterisks denote statistically significant differences between cohorts (*p* < 0.05)

Participants rated 11 aspects of the experience on a 10-point scale, with scores above 7 in both editions except for peer interaction (Fig. 5 and Supplementary Table S1). Mean ratings ranged from 6.0 to 9.4 and were higher in the second edition. Consistent with the Likert-scale results, peer interaction received the lowest ratings. Significant improvements were observed for overall experience (8.0 vs 8.6, *p* = 0.042), project organisation (8.1 vs 9.3, *p* < 0.001), and connectivity (6.6 vs 7.6, *p* = 0.045), while no significant differences were found for the remaining items.

Qualitative analysis of open comments from 47 residents identified recurring themes (Table 4). Positive comments highlighted the teaching methodology, particularly the practical and didactic lectures, workshops, learning resources, and course quality and relevance. Negative comments focused on workload, course organisation, and technical issues affecting the virtual environment. Suggestions addressed course organisation, participant–instructor interaction, expansion of specific content areas, and technical limitations.

**Table 4:** Summary of open comments from the evaluation questionnaire

Comments	Category
**Positive**	
Didactic and practical lectures (14); participation opportunities (12); workshops as the most valued activity (9); availability of resources (5); tasks helped consolidate knowledge (3); pre- and post-tests as learning tools (2)	Teaching methodology and learning design
Foundational contents (17); musculoskeletal lessons (4); enjoyable thorax lessons (3); description of projections and their usefulness (2); focal bone lesion lesson; abdominal block lessons	Course content
Second Life as an interesting teaching methodology (5); worldwide connection possibilities (3); communication agility (2); image resolution adequate for understanding educational content	Platform and learning environment
Excellent teachers (8); thanks, and congratulations (9); willingness to repeat the course (2); opportunity to learn about Second Life; requests to organise more courses	General appreciation
**Negative**	
Too many tasks (8); synchronous review of tasks (5); crowding during sessions (4); on-call flexibility (2); lectures too long; organisation of lectures; too many perception questionnaires; more on-demand content	Workload and course organisation
Shorter course (4); musculoskeletal section somewhat tedious (3); basic thorax anatomy lectures (2); too much information; more musculoskeletal and less thorax; level too high for first-year residents	Course content
Image resolution problems (9); slides skipping (2); difficulty zooming slides	Presentation and slides
Bad connection (5); viewer problems (4); installation restrictions on institutional computers (3); direct login without island navigation (2); audio problems; high technical requirements	Technical issues
**Suggestions**	
Shorter and more numerous lessons; smaller groups per session; block organisation to facilitate studying; monographic versions of the course; reduce the number of tasks; thorax lessons better organised; musculoskeletal block well organised; course level high for first-year residents	Course organisation and structure
More theory on thoracic imaging interpretation; basic resident course on emergency CT and ultrasound; limited teaching of plain radiography in the MIR training programme	Course content
Promote interaction between students and teachers; direct questioning of participants; anonymous responses increase participation	Interaction and participation
Higher image resolution; more screens to perform tasks; some participants not convinced by avatars or the virtual campus	Technical improvements

Positive comments, negative comments, and suggestions were identified through qualitative analysis of participants’ open responses. Numbers in parentheses indicate the number of participants mentioning each comment

## Discussion

This study explored the feasibility of delivering complementary radiology education for residents in a virtual world such as Second Life. Participants evaluated the course positively, considering it appropriate for their training stage and useful for clinical practice, although some perceived the level as somewhat high. Overall, the intervention appeared feasible, although participant retention, peer interaction, task completion, and technical usability remain areas for improvement.

The improvement in pre- and post-course test scores reflects better short-term performance on a repeated knowledge test following course participation. Because identical questions were administered before and after the intervention, these findings should not be interpreted as evidence of durable learning or clinical competence. The lack of significant improvement in the thorax section during the second edition may reflect higher baseline scores, suggesting greater prior knowledge in this group.

This educational format enabled residents from multiple hospitals to participate without travel and provided flexible access through recorded sessions and asynchronous activities. Rapid enrolment in the first edition, which filled within 5 days and generated a waiting list for the second, suggests substantial interest in this complementary educational activity. Previous undergraduate studies reported educational outcomes in virtual worlds comparable to traditional classroom settings [6, 20]. Accordingly, the value of these environments may lie not only in educational outcomes but also in their support for flexible, geographically distributed learning. To our knowledge, this is one of the first studies to evaluate the implementation of this educational approach in postgraduate radiology training, extending previous work in undergraduate medical education.

Participants evaluated the course positively. The virtual-world environment supported synchronous lectures, discussions, and asynchronous educational activities within a shared online space. The current desktop version of Second Life remains freely accessible through a standard computer and internet connection, allowing remote participation without specialised virtual-reality hardware. Educational content, activities, and perceived usefulness for clinical practice received high ratings, and quantitative and qualitative findings were consistent. These results suggest that participants’ positive perceptions were more closely related to the educational content and course organisation than to the immersive characteristics of the platform. Lower ratings for peer interaction, task completion, and some usability-related aspects were also reflected in the open comments, highlighting opportunities to improve interaction dynamics and optimise workload and task design in future editions.

Digital and blended learning are increasingly incorporated into radiology education and have been associated with favourable educational outcomes and participant satisfaction among medical students [21]. In this context, virtual-world courses may provide a complementary educational option for radiology residents, particularly when flexible access and geographically distributed participation are required.

In our study, the musculoskeletal block showed the lowest test and task scores, suggesting greater difficulty during the early stages of residency. Because plain radiography is the initial imaging modality for many musculoskeletal conditions and may be difficult to interpret because of overlapping anatomical structures, reinforcing training in this area may be particularly relevant.

This study has several limitations. First, the absence of a comparator group prevents attributing the observed improvement specifically to the virtual-world environment. In addition, the intervention combined lectures, workshops, asynchronous activities, recorded sessions, and independent study, preventing the determination of the relative contribution of each component or isolation of the virtual-world effect. The same questions were used in the pre- and post-course assessments, and participants were repeatedly exposed to the evaluated content, so teaching-to-the-test and test familiarity effects cannot be excluded. In addition, the 45-item knowledge test had not undergone formal psychometric validation, which may limit the interpretation of the observed score changes. Participation was voluntary and required adequate time, internet connectivity, and computer access, potentially introducing self-selection bias and favouring residents with greater motivation or technical resources. Workload may also have limited participation in extracurricular training. These factors, together with the specific context of the Spanish radiology residency system, may limit the generalisability of the findings. Resource requirements and implementation costs were not formally evaluated and were therefore not included in the feasibility assessment. Finally, long-term learning outcomes were not assessed, and no comparisons were made with face-to-face or conventional teaching formats.

Interaction among participants during clinical sessions was limited despite instructors’ efforts and was one of the lowest-rated aspects of the course. Future editions could incorporate additional collaborative activities to promote interaction. Technical issues have also been reported as barriers to participation in Second Life educational activities [22, 23], with 9–11% of medical students reporting significant difficulties in previous studies [6, 7, 17]. Although these issues may diminish with improved hardware and connectivity, they remain a potential obstacle. Mental effort related to navigation was moderate, but part of the cognitive load may have been attributable to the platform rather than radiology learning, particularly during the initial stages of the course. Finally, developing and implementing the course required considerable time for preparing materials, organising sessions, and designing assignments, a common limitation of Second Life–based educational projects [22, 23]. This burden may be reduced by reusing materials in future editions or related initiatives [24].

Based on our results, virtual worlds may complement postgraduate radiology training, particularly in cohorts composed predominantly of first- and second-year residents and settings with limited access to face-to-face teaching. Their value lies in supporting flexible, geographically distributed learning and access to structured educational activities.

## Conclusions

Our findings suggest that immersive teaching in a three-dimensional virtual world is a feasible approach to radiology resident education. Participation was associated with improved short-term performance on a repeated knowledge test and positive participant perceptions. Despite technical and methodological limitations, this format may complement traditional teaching in cohorts composed predominantly of first- and second-year radiology residents by supporting flexible access to structured educational activities.

## Supplementary information

ELECTRONIC SUPPLEMENTARY MATERIAL

## Data Availability

The datasets generated and/or analysed during the current study are available from the corresponding author on reasonable request, subject to ethical and data protection considerations.

## Abbreviation

SERAM: Spanish Society of Medical Radiology
