# Supplementary material for: A multicentre immersive virtual-world course for radiology residents: feasibility, educational outcomes, and participant perceptions
Source: Insights Imaging. 2026 Oct 29;17:270. doi: 10.1186/s13244-026-02455-6 (PMC13719935; doi:10.1186/s13244-026-02455-6)
Supplement: Supplementary file 1 [file 13244_2026_2455_MOESM1_ESM.pdf]

A multicentre immersive virtual-world course for radiology residents: feasibility, educational outcomes, and participant perceptions

ELECTRONIC SUPPLEMENTARY MATERIAL

Insights Imaging (2026) Pino-Postigo A, Domínguez-Pinos D, Navarro-Sanchis EL, Ruiz Gómez MJ, Sendra-Portero F

Supplementary Table S1: Item-level results of the post-course evaluation questionnaires completed after the 2021 and 2022 course editions.

5-point Likert scale*<br>Items	1st edition 2021 (n = 25)<br>1	2	3	4	5	2nd edition 2022 (n = 34)<br>1	2	3	4	5	*P value*
About Second Life											
**1. The initiative seemed interesting to you**	0.0	0.0	4.0	28.0	68.0	3.0	0.0	3.0	21.2	72.7	*0.767*
**2. The island environment seemed attractive to you**	0.0	4.0	32.0	48.0	16.0	3.0	3.0	24.2	18.2	51.5	*0.073*
**3. The postgraduate building seemed suitable for taking the course**	0.0	0.0	16.0	56.0	28.0	0.0	6.1	3.0	33.3	57.6	*0.041*
**4 You can navigate Second Life easily, without problems**	8.0	8.0	44.0	28.0	12.0	9.1	9.1	18.2	48.5	15.1	*0.252*
**5. The tasks of creating and managing your avatar were easy**	4.0	16.0	36.0	28.0	16.0	6.1	12.1	18.2	39.4	24.2	*2.265*
**6. Your computer allows you to work in Second Life without problems**	12.0	20.0	24.0	20.0	24.0	3.0	18.2	27.3	27.3	24.2	*0.468*
**7. Your Internet connection allows you to work in Second Life without problems**	4.0	8.0	24.0	28.0	36.0	0.0	15.2	15.2	30.3	39.4	*0.761*
**8. You would be willing to participate in another experience in Second Life**	0.0	8.0	16.0	32.0	44.0	0.0	3.0	18.2	21.2	57.6	*0.368*
About the course											
**9. The course seemed appropriate for your training**	0.0	0.0	8.0	32.0	60.0	0.0	0.0	3.0	24.2	72.7	*0.285*
**10. The contents seemed useful for clinical practice**	0.0	0.0	0.0	32.0	68.0	0.0	0.0	6.1	21.2	72.7	*0.828*
**11. The scheduling seemed appropriate**	0.0	0.0	16.0	48.0	36.0	0.0	0.0	3.0	54.5	42.4	*0.325*
**12. The format of synchronous lectures is useful for your training**	0.0	0.0	12.0	56.0	32.0	0.0	0.0	3.0	36.4	60.6	*0.024*
**13. You were able to follow the presentations easily**	0.0	8.0	16.0	44.0	32.0	0.0	0.0	18.2	48.5	33.3	*0.660*
**14. The format of tasks and virtual workstations is useful for your training**	4.0	12.0	20.0	48.0	16.0	6.1	9.1	12.1	24.2	48.5	*0.072*
**15. The tasks were interesting**	4.0	4.0	4.0	52.0	36.0	0.0	6.1	15.2	27.3	51.5	*0.535*
**16. The virtual workstations were interesting**	0.0	0.0	20.0	52.0	28.0	0.0	9.1	18.2	18.2	54.5	*0.331*
**17. You were able to complete the tasks and work with virtual workstations easily**	16.0	20.0	44.0	20.0	0.0	3.0	15.2	21.2	33.3	27.3	*0.002*

**18. Your participation in the course was very active**	4.0	8.0	40.0	32.0	16.0	0.0	21.2	39.4	27.3	12.1	*0.042*
**19. You interacted with other classmates**	16.0	24.0	32.0	24.0	4.0	18.2	33.3	27.3	9.1	12.1	*0.560*

**0-10 rating scale** Items**	**1st edition 2021 (n = 25)**	**2nd edition 2022 (n = 34)**	***P value***
**1. Overall experience**	8.0 ± 0.8	8.6 ± 1.2	*0.042*
**2. Project organization**	8.1 ± 1.1	9.3 ± 1.0	*< 0.001*
**3. The island environment**	7.6 ± 1.2	8.1 ± 1.6	*0.124*
**4. Educational content**	8.6 ± 1.1	9.0 ± 0.9	*0.170*
**5. Usefulness for your training**	8.8 ± 1.2	9.0 ± 1.1	*0.539*
**6. The instructors**	9.0 ± 0.8	9.4 ± 0.7	*0.070*
**7. Interaction with classmates**	6.0 ± 2.1	6.4 ± 2.2	*0.399*
**8. Synchronous sessions**	7.8 ± 1.3	8.4 ± 1.2	*0.081*
**9. The tasks**	7.4 ± 1.7	7.8 ± 2.2	*0.360*
**10. The virtual monitors**	7.4 ± 1.2	7.6 ± 2.2	*0.794*
**11. Connectivity to Second Life**	6.6 ± 2.2	7.6 ± 1.6	*0.045*

*Data are presented as percentages of responses on a 5-point Likert scale: 1 = Totally disagree; 2 = Disagree; 3 = Neither agree nor disagree; 4 = Agree; 5 = Totally agree. *P* values were calculated using the Mann–Whitney U test.

**Data are presented as mean ± standard deviation (SD). *P* values were calculated using the unpaired Student's *t* test.
